# Τhiazolidine-4-One Derivatives with Variable Modes of Inhibitory Action Against DPP4, a Drug Target with Multiple Activities and Established Role in Diabetes Mellitus Type II †

**DOI:** 10.3390/ph18010052

**Published:** 2025-01-04

**Authors:** Dionysia Amanatidou, Phaedra Eleftheriou, Anthi Petrou, Athina Geronikaki, Theodoros Lialiaris

**Affiliations:** 1 Department of Biomedical Sciences, School of Health, International Hellenic University, 57400 Thessaloniki, Greece; damanatidou@ihu.gr; 2 Department of Pharmaceutical Chemistry, School of Pharmacy, Aristotle University of Thessaloniki, 54124 Thessaloniki, Greece; aipetrou@pharm.auth.gr (A.P.); geronik@pharm.auth.gr (A.G.); 3 School of Medicine, Democritus University of Thrace, 68100 Alexandroupolis, Greece; lialiari@med.duth.gr

**Keywords:** DPP4, competitive inhibitors, non-competitive inhibitors, uncompetitive inhibitors, mixed inhibition, allosteric center, docking analysis, probability factor for binding PF, 3-(benzo[d]thiazol-2-yl)-2-aryl thiazolidin-4-ones, diabetes mellitus type II

## Abstract

**Background/Objectives:** DPP4 is an enzyme with multiple natural substrates and probable involvement in various mechanisms. It constitutes a drug target for the treatment of diabetes II, although, also related to other disorders. While a number of drugs with competitive inhibitory action and covalent binding capacity are available, undesired side effects exist partly attributed to drug kinetics, and research for finding novel, potent, and safer compounds continues. Despite the research, a low number of uncompetitive and non-competitive inhibitors, which could be of worth for pharmaceutical and mechanism studies, was mentioned. **Methods:** In the present study sixteen 3-(benzo[d]thiazol-2-yl)-2-aryl thiazolidin-4-ones were selected for evaluation, based on structural characteristics and docking analysis and were tested in vitro for DPP4 inhibitory action using H-Gly-Pro-amidomethyl coumarin substrate. Their mode of inhibition was also in vitro explored. **Results:** Twelve compounds exhibited IC_50_ values at the nM range with the best showing IC_50_ = 12 ± 0.5 nM, better than sitagliptin. Most compounds exhibited a competitive mode of inhibition. Inhibition modes of uncompetitive, non-competitive, and mixed type were also identified. Docking analysis was in accordance with the in vitro results, with a linear correlation of logIC_50_ with a Probability of Binding Factor(PF) derived using docking analysis to a specific target box and to the whole enzyme. According to the docking results, two probable sites of binding for uncompetitive inhibitors were highlighted in the wider area of the active site and in the propeller loop. **Conclusions:** Potent inhibitors with IC_50_ at the nM range and competitive, non-competitive, uncompetitive, and mixed modes of action, one better than sitagliptin, were found. Docking analysis was used to estimate probable sites and ways of binding. However, crystallographic or NMR studies are needed to elucidate the exact way of binding especially for uncompetitive and non-competitive inhibitors.

## 1. Introduction

Dipeptidyl-peptidase 4 (DPP4 or CD26) is a widely studied biomolecule involved in a variety of processes [1]. It is found on the surface of most cells, while also exists in soluble form. It acts both as a receptor and as a serine exopeptidase detaching an amino-terminal dipeptide by cleaving next to proline or alanine [2,3,4].

Currently, DPP4 is a drug target for the treatment of diabetes type II, because of its involvement in the hydrolysis of the incretins GLP-1 (glucagon-like peptide-1) and GIP (glucose-dependent insulinotropic polypeptide) which induce glucose-dependent insulin secretion and are related to β-cells protection and proliferation [5,6]. DPP4 inhibitors are a class of anti-diabetic drugs, first approved in 2006 [7].

Experimental data also proved the involvement of DPP4 in biochemical pathways, related to immune regulation, inflammation, signal transduction, and apoptosis [1]. Since neuropeptides, such as NPY, NYY, and substance P, are among their substrates, DPP4 activity may affect their function in CNS and peripheral tissues. NPY, known for its anxiolytic activity [8], has been related to the regulation of energy balance, memory, and learning, and low levels of this neuropeptide are found in patients with depression [9]. Therefore, DPP4 could be a possible therapeutic target for depression, anxiety, and mental disorders, as well [9,10].

In addition, DPP4 expression and activity have been related to cancer development with controversial results. CD26+ cancer stem cells have been found and the role of DPP4 in the development of metastases has been highlighted. Altered DPP4 activity has been correlated with numerous tumors [11] and administration of approved DPP4 inhibitors to experimental animals appeared to limit colon cancer or lung metastasis [12]. However, other studies indicated a probable role of DPP4 inhibitors in cancer progression, proposing malignancies as one of the undesired side effects of DPP4 inhibitors [13,14,15,16].

Although a number of DPP4 inhibitors have been approved and have been in use for the treatment of Diabetes type II, for over a decade, the research for finding novel potent DPP4 inhibitors continues mainly because of the accumulated data concerning the side effects and the need for safer members of this drug class. Among the ten most frequent side effects of DPP4 inhibitors are gastrointestinal nonspecific inflammation, hypersensitivity, acute pancreatitis, haemodynamic oedema, malignancies, angioedema, embolic/thrombotic events, hepatic disorders, and cardiac failure [17]. The mechanism underlying these effects is not clear. However, increased levels of DPP4 substrates due to inhibition of its activity have been proposed in most cases [18,19]. Although the side effects characterize the drug class, differences between specific drugs are attributed mainly to differences in drug kinetics [19].

The multiplicity of natural substrates of DPP4, with 40 different molecules found till now [20,21,22,23,24,25,26,27,28,29] may explain the involvement of DPP4 in multiple pathways and the variety of side effects. The effect of DPP4 on its substrates may result in an inactive product but may also lead to an alteration in the activity, specificity, and half-life of the initial substrate [30,31,32], sometimes leading to a more specific, more stable product [32].

As an enzyme with a crucial role in several mechanisms, the action of DPP4 must be under strict control by factors some of which may be unknown till now, and allosteric sites for the binding of natural inhibitors or enhancers are expected to be revealed.

Currently approved DPP4 drugs bind at the active site of the enzyme acting as competitive inhibitors (sitagliptin, alogliptin) or form covalent bonds with the catalytic amino acid which leads to prolonged inhibition (saxagliptin, vildagliptin) [33,34]. Competitive inhibitors have reversible activity, exhibiting increased inhibition at lower substrate concentrations and low to no efficiency at relatively high substrate concentrations, which may be present at a tissue level under specific circumstances. In the case of enzymes with multiple substrates, the high cumulative concentration of different substrates may be observed locally, especially if the substrates are products of the same precursor-protein, they are co-secreted or one acts as a secretion enhancer of the other and they are increased simultaneously in response to the same stimuli. These kinds of interactions occur in the group of DPP4 insulinotropic substrates [35]. At the same time, abnormal increase of other enzyme substrates, secreted at low concentrations at distant locations can be observed. On the other hand, covalent bond-forming inhibitors may cause prolonged inhibition which could also lead to abnormal increase in DPP4 substrates with probable side effects. Inhibitors with non-covalent, reversible binding to allosteric sites could cause more balanced inhibition at a wide range of substrate concentrations. Moreover, the discovery of such inhibitors may constitute the first step for finding allosteric sites of the enzyme and better exploring its role and interactions. Till now, a very limited number of studies refer to such inhibitors [36,37,38,39,40,41,42,43].

In the present study, we aim to discover novel, potent DPP4 inhibitors focusing on finding molecules with different modes of inhibitory action from the traditional competitive inhibitors. For this reason, sixteen thiazolidine-4-one derivatives were selected for evaluation, based on their structural characteristics and the prediction results of the docking analysis.

The known DPP4 inhibitors are molecules rich in N atoms, containing at least one CO group bound to N, in a peptide bond mimicking effort. Aromatic and hydrophobic groups, among which phenyl (e.g., sitagliptin, alogliptin) and adamantane (e.g., saxagliptin, vildagliptin) moieties are present in the structures. Fluorine atoms, mostly in the form of F-substituents of phenyl rings or -CF_3_ groups, are present in several approved inhibitors such as sitagliptin, gemigliptin, and evogliptin. In the studied compounds, the central thiazolidine-4-one moiety contains the CO-N group present in all DPP4 inhibitors, while the thiazolyl ring enriches the N presence in the molecule. The phenyl rings surrounding the central moiety contribute to the hydrophobic and aromatic characteristics of the molecules, while adamantane is an important substituent of three of the compounds. Halogen atoms are present in all structures with F-atoms being present as phenyl ring substituents or as part of -OCF_3_ groups in eleven out of the sixteen selected compounds.

The compounds were tested in vitro for DPP4 inhibitory action and the mode of inhibition was explored. In vitro experiments revealed the existence of competitive, non-competitive, uncompetitive, and mixed inhibitors among the tested compounds.

## 2. Results

### 2.1. Selection of Compounds—Molecular Docking Prediction of Inhibitory Action

**Figure 1:**
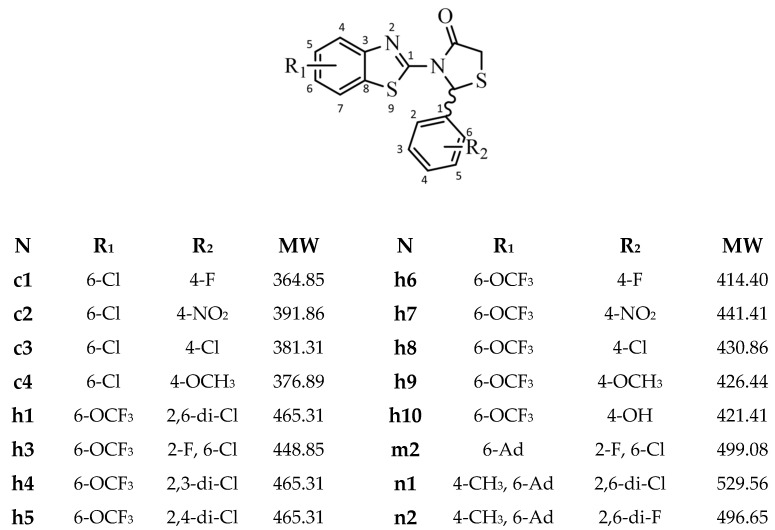
Structure of studied compounds.

Sixteen 3-(benzo[d]thiazol-2-yl)-2-aryl thiazolidin-4-one derivatives (Figure 1) previously synthesized [44,45,46,47] and available for evaluation, were selected as promising candidates for DPP4 inhibitory action since the structure of most derivatives exhibited characteristics and 3D structure potential arrangement resembling the structure and probable binding of known inhibitors of the enzyme. The compounds are structurally divided into three subgroups according to the R1 substituent of the benzo[d]thiazol-2-yl moiety (Figure 1). These are the c-group with a 6-Cl substituent, the h-group with a 6-OCF_3_ substituent, and the m/n group with a 6-Ad substituent.

The probability of stable binding of the compounds at the active site or any other site of the enzyme was estimated using docking analysis.

For docking analysis, a 3D structure of the enzyme in complex with a competitive inhibitor, derived from the Protein Data Bank (2OAG) was used. Although a restricted number of inhibitors with a mode of action indicating the existence of a probable allosteric site have been mentioned in the literature [36,37,38,39,40,41,42,43], no crystallographic or NMR analysis of such inhibitors has been published and the probable allosteric site remains unknown.

Docking analysis using an enzyme structure which was crystallized in a complex with a competitive inhibitor is ideally used to predict binding at the active site because of the ability of the enzymes to slightly change their conformation when complexed with a substrate or an inhibitor. A more accurate prediction is obtained when the complexed inhibitor at the crystallized enzyme form (initial ligand) resembles the studied compounds in magnitude or even in structure [48]. As the initial ligand in 2OAG has a magnitude resemblance to the studied compounds, prediction results were expected to have adequate accuracy for competitive inhibitors.

For the prediction of competitive inhibition, the active site was selected as a docking target surrounded by a docking box of 20 × 20 × 20.

In addition, docking analysis using the whole enzyme as a docking target was performed in order to evaluate the existence of sites which could serve as allosteric sites in the case of inhibitors that do not have a competitive mode of inhibitory action.

Docking to the whole enzyme in parallel with docking targeted to the active site has been proposed for better prediction results even when searching for competitive inhibitors [49]. In this case, in compounds which strongly bind at the active site, the probability of acting as potent competitive inhibitors is reduced if there are other sites of stronger binding potential (lower Estimated Binding Energy in docking results). In such cases, the experimental IC_50_ value tends to be much higher than the predicted one.

For both reasons, docking analysis was performed setting as docking target the active site or the whole enzyme. The results are presented in Table 1.

As shown in Table 1, the Estimated binding Energy varied between −6.46 and −11.04 kcal/mol. According to previous results using various enzymes [48,49], these results are predictive of active inhibitors.

The results revealed that compounds of the group **h** (R1: 6-OCF_3_) with the exception of compound **h10** and group **m/n** (R1: 6-Ad or 4-CH_3_, 6-Ad) exhibited lower Estimated Binding Energy and thus a higher probability of strong inhibitory action compared to compounds of the group-**c**.

Interestingly, in several cases, when the whole enzyme was used as a drug target, a lower Estimated Binding Energy was produced for binding to sites different from the active site. This may indicate that an allosteric site for the binding of allosteric inhibitors exists or may indicate the presence of a strong binding site with no effect on the catalytic activity of the enzyme. This will antagonize binding at the active site, reducing the potency of predicted competitive inhibitors.

In vitro evaluation of inhibitory potency is the only way to confirm inhibition and evaluate the mode of inhibitory action. According to the docking analysis, all compounds were of worth for in vitro evaluation.

### 2.2. In Vitro Evaluation of Inhibitory Action

The compounds were screened for their inhibitory activity against DPP4 by a fluorometric assay, measuring the hydrolysis rate of the substrate Gly-Pro-amidomethyl coumarin in the absence and presence of the inhibitor. The initial screening was conducted at a compound concentration of 80 nM (for the compounds of groups **h**, **m/n**) or 10 μM (for the compounds of group **c**) in two substrate concentrations: one close to the Km value (50 μM) and a second significantly higher. This approach allowed a preliminary evaluation of the mode of inhibitory action, as competitive inhibitors exhibit significantly lower inhibition potency at high substrate concentrations, while uncompetitive inhibitors do the opposite and no or low effect of substrate concentration is observed in non-competitive or mixed inhibitors, respectively.

As shown in Table 2, all compounds demonstrated notable inhibition with twelve of the compounds expected to exhibit IC_50_ values at the nanomolar range and four of the compounds expected to have IC_50_ values at the micromolar range. Two of the compounds exhibited higher inhibition potency at higher substrate concentration (bold) which is characteristic of uncompetitive inhibitors and one of the compounds showed about equal inhibition at both substrate concentrations (bold), which is indicative of non- competitive or mixed inhibitors.

**Figure 2:**
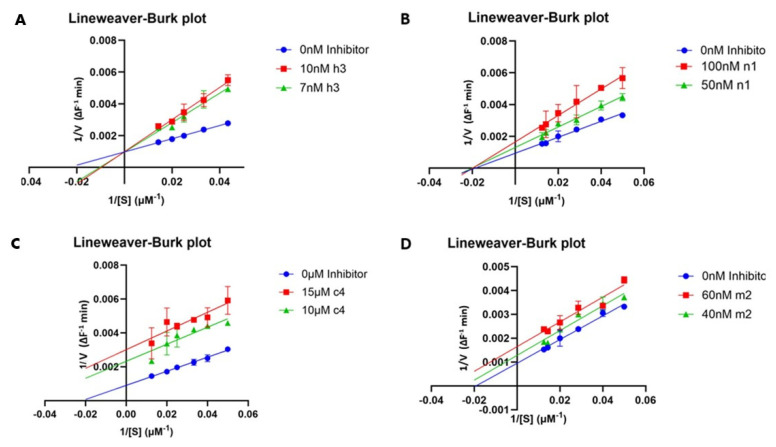
Lineweaver–Burk blots for the compounds **h3** (**A**), **n1** (**B**), **c4** (**C**), and **m2** (**D**). As shown by the curves, the modes of inhibitory action are competitive for **h3**, non-competitive for **n1**, and uncompetitive for **c4** and **m2**.

The potency was further explored by the determination of the IC_50_ values of the compounds (Table 3). For determination of the mode of inhibitory action, the Lineweaver–Burk plots in the absence and presence of the compounds were prepared (Figure 2).

### 2.3. Inhibition Kinetics

To further investigate the mode of inhibition, Lineweaver–Burk curves were prepared for some of the compounds. The studied compounds include the most active compounds of each group and the compounds for which the preliminary experiment at two substrate concentrations (Table 2) indicated a mode of action different from competitive which is common for most published inhibitors and the approved drugs.

The Km and Vmax for the compounds **h3**, **h4**, **h9**, **m2**, **n1**, **n2**, **c2** and **c4** are shown in Table 4. Indicative Lineweaver–Burk curves for the compounds **h3**, **m2**, **n1,** and **c4** are shown in Figure 2.

The Km and Vmax values, determined using the Lineweaver–Burk blots, confirmed the preliminary results.

Compounds **h3**, **h4**, **c2**, and **n2** exhibited, as expected, characteristics of competitive inhibitors, since Vmax remained unchanged, while Km increased. Compounds **c4** and **m2** exhibited characteristics of uncompetitive inhibitors as both Vmax and Km decreased while **n1** showed a decrease in Vmax but no change in Km which indicates non-competitive inhibition. Compound **h9** showed a mixed inhibition mode.

### 2.4. Docking Analysis

**Figure 3:**
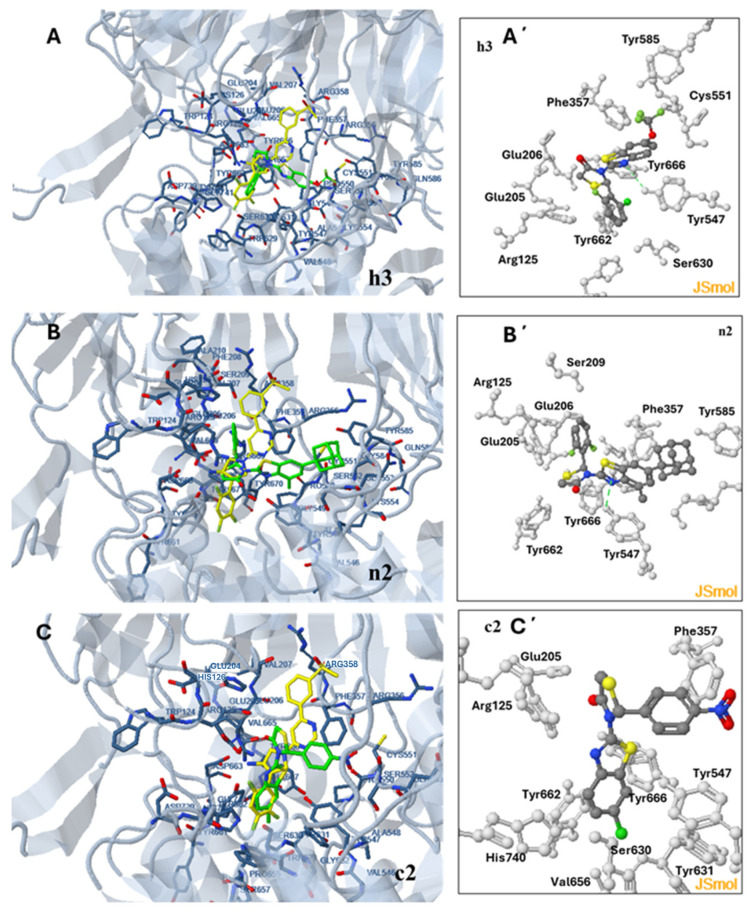
Docking of the competitive inhibitors **h3** (**A**,**A′**), **n2** (**Β**,**Β′**) and **c2** (**C**,**C′**) in the active site of DPP4. The docked compound is shown in green. The initial ligand is shown in yellow.

According to the docking analysis, the orientation of competitive inhibitors at the active site of the enzyme differentiates depending on the structure of the molecule. However, the most stable orientations share common characteristics. In the case of compounds **h3**, **n2,** and **c2**, shown in Figure 3, the thiazolidinone ring is placed at about the same position interacting with Glu206 in all three cases and with Tyr666 in the case of **h3** and **n2.** This is the position occupied by the pyrrolidine ring of the initial ligand of the DPP4 structure.

In the case of **h3**, the benzo[d]thiazol-2-yl moiety is oriented towards Tyr585 and Cys551 with which, the -OCF_3_ group forms halogen bonds, while the benzene and thiazole groups form pi interactions with Phe357 and Tyr547 surrounding them. The orientation is also stabilized by a hydrogen bond formed between the H of the OH group of Tyr547 and the N atom of the thiazolyl ring. The 2F,6Cl phenyl ring is oriented towards Tyr662 with which it forms halogen bonds, while the complex is stabilized by hydrophobic interactions between Tyr666 and the thiazolidine-4-one ring.

A similar orientation is adapted by other competitive inhibitors of the **h**-group with slight differentiation due to the different substituents.

Compound **n2**, adapts a different orientation. The benzo[d]thiazol-2-yl moiety is also oriented towards Tyr585 and Gln553 which form hydrophobic interactions with adamantane. However, the 2,6-di-F phenyl ring is directed towards Glu205 and Glu206 with which it forms halogen bonds. Hydrophobic interactions between the methyl substituent and the amino acids Phe357, Tyr547, and Tyr666 also stabilize the complex along with pi–pi interactions of the carbon atoms of the benzyl ring with Phe357 and Tyr547.

The orientation of compound **c2** differentiates from that of competitive inhibitors of the **h** and **n** group in the orientation of the benzo[d]thiazol-2-yl moiety which is now directed towards Val656 and Ser630 in an area surrounded by Tyr631, Tyr547, Tyr662 and His740. Pi–pi interactions of the p-nitro-phenyl ring with Phe357 and Tyr547 significantly contribute to complex stabilization. Tyr666 also participates in pi–pi interactions with the benzyl ring of the benzo[d]thiazol-2-yl moiety. Polar interactions are formed between Arg122 and the thiazolyl and thiazolidine-4-one groups, while a hydrogen bond between Glu205 and the thiazolidine-4-one group also stabilizes the complex.

**Figure 4:**
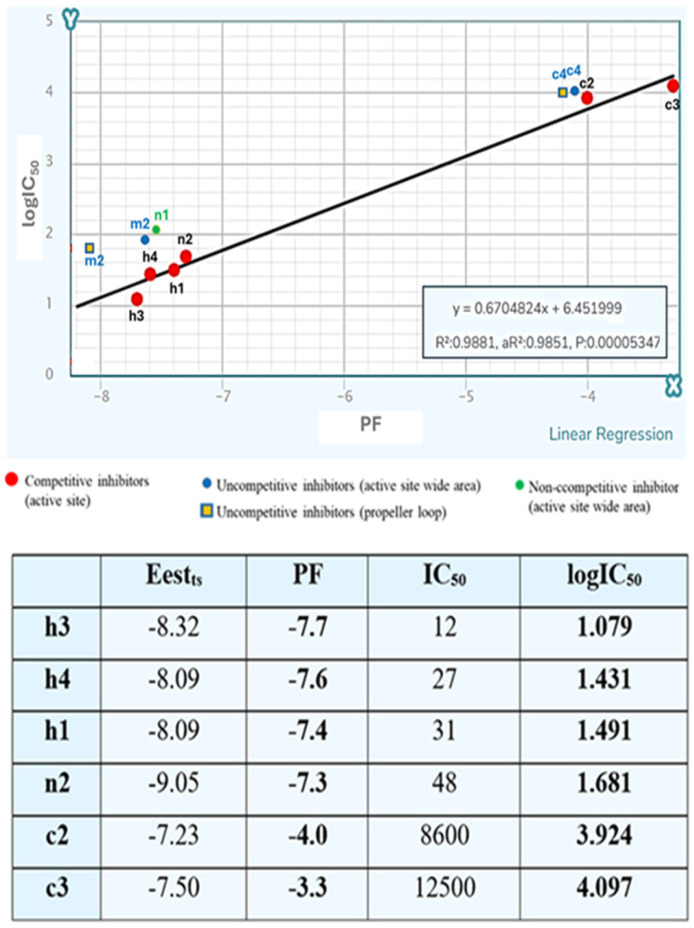
Correlation of log IC_50_ of competitive inhibitors with the Probability Factor (PF). Only competitive inhibitors were taken into account for the estimation of linear regression. The PF is calculated if we modify the Eest exported from docking to the target site box (Eest_ts_), which corresponds to the active site for competitive inhibitors, by abstracting a factor (d) produced using the results of docking to the whole enzyme at all positions (x) with lower binding energy (Eest_x_) than that of the target site (Eest_t_) which is the active site for competitive inhibitors. Factor d = $\sum (\Delta Ex*\frac{\nu x}{100})*10$, where ΔΕx = Est_x_ − Eest_t_ and v_x_ is the frequency (%) of binding to the specific site x with the specific pose which corresponds to Estimated binding Energy Est_x_.

In general, **c2** exhibits decreased options for halogen bond formation and hydrophobic interactions compared to **h3** and **n2** and this may explain the higher Estimated Binding Energy (Eest) calculated by the docking program (−7.23 kcal/mol compared to −8.32 and −9.05 Kcal/mol of **h3** and **n2**, respectively). However, the difference in in vitro determined IC_50_ values of the compounds is only partially explained by the calculated Eest exported by the docking to the active site. Application to the whole enzyme indicated the probability of binding to other sites apart from the active site for all the compounds, a commonly occurring situation. A higher number of such sites was revealed for the much less potent compound **c2** compared to **h3** and **n2**. If these binding sites are taken into account, the in vitro determined IC_50_ correlates well with the prediction results. An algorithm exported by docking to both the active site and the whole enzyme, leading to the calculation of a Probability Factor (PF), explains this correlation as shown in Figure 4.

Although the number of competitive inhibitors is small, the predicted PF follows a linear regression with logIC_50_ with R^2^ = 0.9881, and *p* = 0.00005347 which is an encouraging indication of the reliability of docking results. Calculation of PF substantially improves the correlation between docking results and in vitro determined IC_50_. Correlation of the Eest calculated by docking to the target site box (Eest_ts_) with logIC_50_ exported much poorer linear regression results with R^2^ = 0.5932 and *p* = 0.07316.

In all cases, docking results are only indicative and can be used with many restrictions to predict activity and mode of inhibition.

Uncompetitive (**m2**, **c4**) and non-competitive (**n1**) inhibitors of this study gather more interest, since such inhibitors are rarer and may have promising applications. The existence of such inhibitors indicates the presence of an allosteric site with which they bind. Crystallographic and NMR studies must be conducted in order to specify the position of this site.

An effort to predict the probable position of the allosteric site was conducted using the results of the docking analysis of the whole enzyme. This site can be within the cavity of the active site or can be outside the cavity at a neighboring or remote location. There are many examples in which the allosteric site is placed at a remarkable distance. PTP1b is one such enzyme [50].

In the case of DPP4, an analog phenomenon of remote control of enzyme activity exists concerning mutations of amino acids at positions far away from the active site with no obvious implication in catalysis. Despite the distance, the mutations affect the Κm and Vmax of the enzyme. Such mutations are T351A, K554Q, R492K, V486M, D65E, and A291P, with V486M which leads to inactivation, being the most remote one [51]. This indicates that in this enzyme, an effect at a site at a great distance from the catalytic triad may cause inhibition and the sites of inhibiting mutations may be such sites.

The existence of multiple binding sites turns the exploration of a possible allosteric site into a more complicated process. Since certain sites are also occupied by competitive inhibitors with estimated binding Energies lower than that of active sites, binding to these sites does not cause inhibition. Under this concept, the low energy binding sites of competitive inhibitors were excluded to reveal the possible sites where the uncompetitive or non-competitive inhibitors bind and could be correlated with inhibitory action.

**Figure 5:**
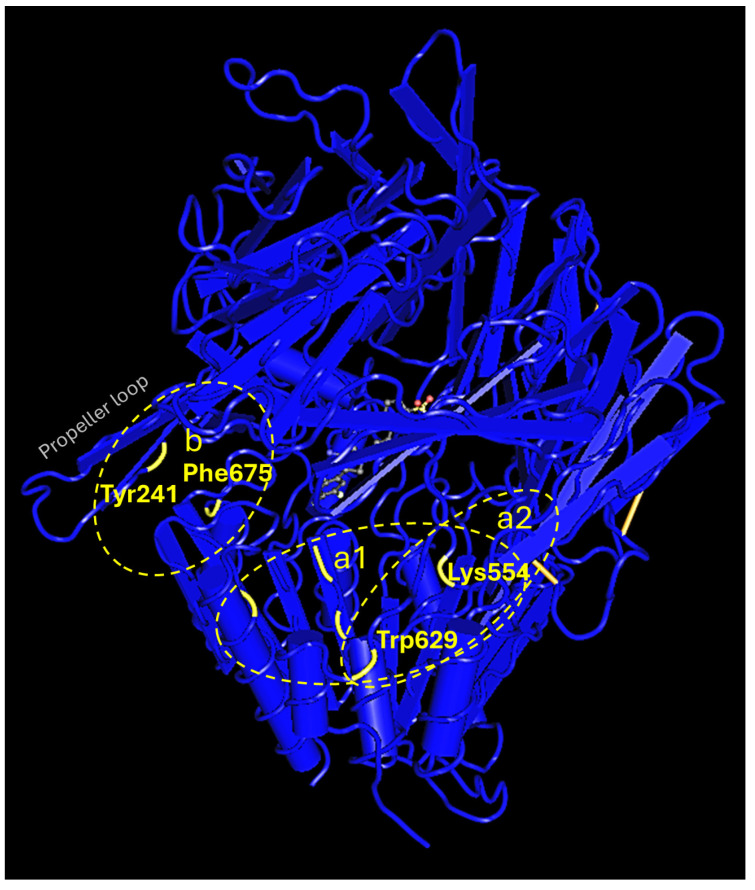
Probable sites of binding of uncompetitive (sites a1, b) and non-competitive (site a2) inhibitors.

**Figure 6:**
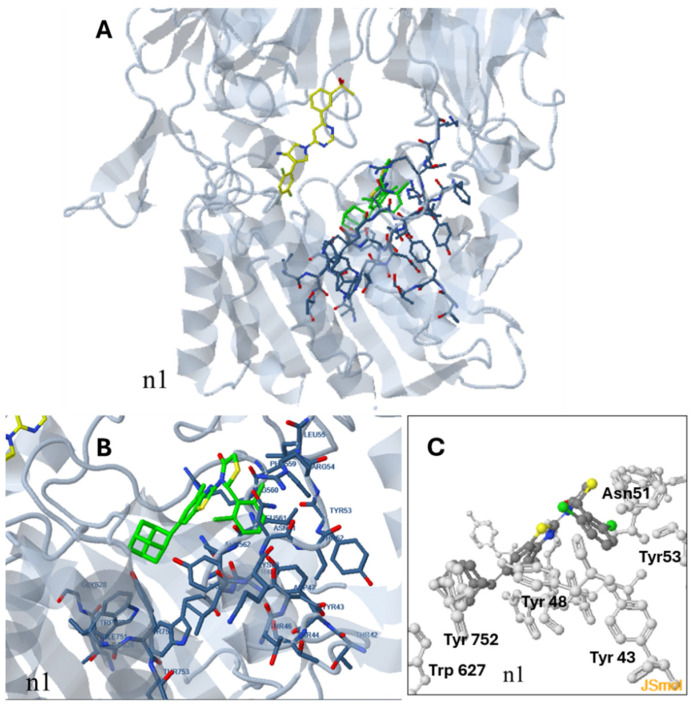
Probable binding site (site a2) of the non-competitive inhibitor **n1**. (**A**) shows the orientation of n1 (in green) within the active site in relation to a competitive inhibitor (initial ligand of the structure, in yellow). The amino acids participating in interactions with **n1** are shown in (**B**,**C**).

**Figure 7:**
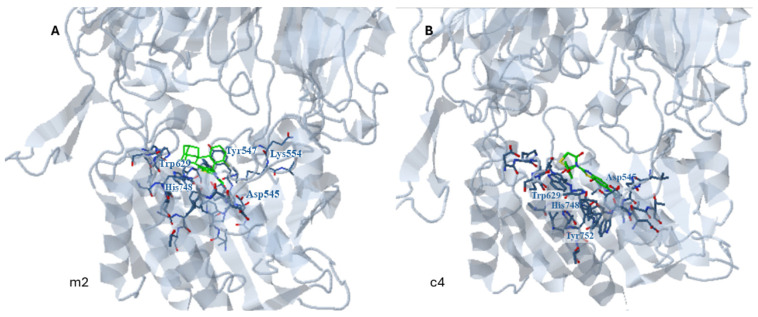
Probable site of binding (site a1) of the uncompetitive inhibitors **m2** (**A**) and **c4** (**B**) within the active site of DPP4. The docking was applied to the whole enzyme in the presence of the initial ligand.

**Figure 8:**
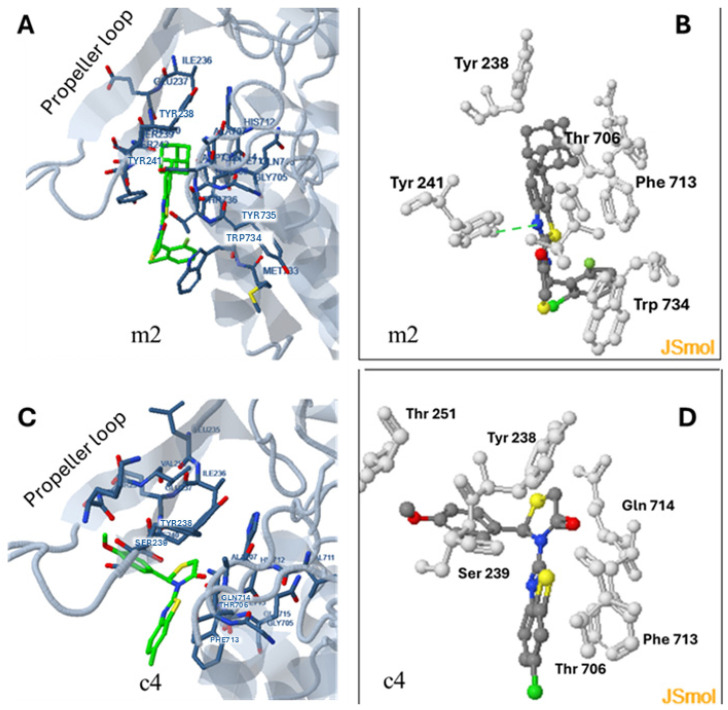
Docking of the uncompetitive inhibitors **m2** (**A**,**B**) and **c4** (**C**,**D**) at the probable **site b**, between the propeller loop (residues 234–260) and the residues around Phe713 and Thr706 near the catalytic triad (Ser630, Asp708, His740) of the enzyme.

The exclusion-process exported two probable sites of binding: (a) a site (a1 and a2) within the cavity of the active site (Figure 5, Figure 6 and Figure 7) and (b) a site at the propeller loop (Figure 5 and Figure 8).

For compound **n1** which acts as a non-competitive inhibitor, docking analysis indicated as most probable binding site, a site within the cavity of the active site at a distant area from the ligand (**site a2**) near the amino acids Tyr752, Phe559, Asn51, and Tyr48 (Figure 6).

According to the docking analysis, compound **n1** is oriented with the 2,6-dicloro-phenyl group towards Tyr48 and Tyr53 with which it forms halogen bonds and the adamantane moiety towards Tyr752 with which it forms hydrophobic interactions. Hydrophobic interactions are also formed between Trp627 and the adamantane group and between Leu561 and the 2,6-dicloro-phenyl group. The complex is further stabilized by pi–pi interactions between Tyr48 and the benzyl ring of the benzo[d]thiazol-2-yl moiety as well as the 2,6-dicloro-phenyl moiety (Figure 6).

As uncompetitive inhibitors preferentially bind at the enzyme—substrate complex, docking to the whole enzyme with the initial competitive inhibitor kept at the enzyme structure is expected to better reveal probable binding sites of such inhibitors with effect in catalysis. 

Figure 7 shows the orientation of compounds **m2** and **c4** with uncompetitive inhibitory action in the **area a1** of the active site of the enzyme when docking was applied to the whole enzyme in the presence of initial ligand (structure 2OAG complexed with DLI B).

As expected, when bound at the active site in the presence of the inhibitor, the compound is oriented near the entrance of the cavity.

In the case of compound **m2**, hydrophobic interactions with Tyr547 and His748 mostly contribute to complex stabilization followed by halogen bond formation with Ser630. Pi-pi interactions with Tyr547 also stabilize the complex, while interactions with Ala743, Asp545, and Lys554 are also observed.

In the case of compound **c4**, hydrophobic and pi–pi interactions with Trp629, Trp627, and Ala743 are observed with significant contributions to complex stabilization. A hydrogen bond is formed between His 748 and the thiazolyl ring of the benzo[d]thiazol-2-yl moiety while As545 participates in a halogen bond with the Cl atom of the phenyl ring.

The Probability Factor for probable binding to site a1, calculated based on the Estimated Binding Energy correlated well with the IC_50_ of compounds **m2** and **c4**, roughly following the equation produced by the competitive inhibitors (Figure 4).

Similar results were obtained when compounds **m2** and **c4** were docked at the (Tyr-Pro-Ser)–DPP4 simulated by the docking program. The tripeptide constitutes the amino-terminal peptide of a natural substrate of DPP4.

The binding of the uncompetitive inhibitors at **site b**, between the propeller loop (residues 234–260) and the residues around Phe713 and Thr706 is near enough to the catalytic triad (Ser630, Asp708, His740) to affect catalytic activity; however, binding at this site is restricted (Figure 8). DPP4 forms a dimer with the propeller loop taking part in dimer formation. DPP4 dimers are not in equilibrium with monomers and dimerization is necessary for catalytic activity [51,52]. Interestingly, during catalysis, the propeller loop moves several times between a “closed” and an “open” pose, and this movement is considered as essential for the catalytic procedure. Preventing propeller loop movement in V486M mutation leads to the inactivation of the enzyme [51]. We do not know if the conformations adapted during the movement of the loop enable the binding of the inhibitors.

The docking results, obtained by docking the compound at the monomer, indicated a fair correlation between the calculated PF, produced for probable binding at site **b**, and the determined IC_50_ values of compounds **m2** and **c4** (Figure 4).

According to the docking results, Tyr238 and Tyr241 are the main amino acids of the propeller loop that contribute to complex stabilization with Tyr238 being mostly involved in hydrophobic and pi–pi interactions with adamantane in the case of **m2** or with the 4-HO-phenyl moiety in the case of **c4**. Additionally, Tyr241 participates in hydrogen bond formation with the N of the thiazolyl group in the case of **m2** and in halogen bond formation in the case of the **c4** inhibitor. Phe713 dominates among the amino acids outside the loop in contribution to complex stabilization via hydrophobic and pi–pi interactions in the case of both inhibitors. The hydrophobic adamantane group of **m2** favors inhibition affecting the orientation of the molecule and the number of hydrophobic interactions formed (Figure 8).

### 2.5. Prediction of Toxicity of the Compounds

Among the studied compounds, the most potent inhibitors could be considered as drug candidates or leading structures for the synthesis of novel drugs for the treatment of Diabetes type II. Under this option, the potential toxicity of the compounds must be studied. In the present study, a theoretical prediction of toxicity was performed using the pkCSM and ProTox 3 Programs, predicting Small-Molecule Pharmacokinetic and Toxicity Properties. Oral Rat Acute Toxicity (LD_50_) was calculated, along with CaCo2 cell permeability and Human Intestinal Permeability (HIA) values. The organ-specific toxicities hepatotoxicity, nephrotoxicity, cardiotoxicity, as well as carcinogenicity which have been associated with approved DPP4 inhibitors were also evaluated [17]. In addition, prediction of the probable hepatic metabolism of the compounds was performed by estimating their ability to act as substrates of the most important hepatic enzymes, also involved in the metabolism of known gliptins. The prediction results are shown in Table 5. Sitagliptin, Linagliptin, Alogliptin, Saxagliptin, and Vildagliptin were used for comparison reasons.

According to the prediction results, the compounds are not expected to show increased acute toxicity. Most of them are expected to be less toxic than sitagliptin and saxagliptin. In addition, most of them and especially the most potent ones are not expected to exhibit organ specific toxicity or carcinogenicity.

## 3. Discussion

Twelve out of the sixteen 3-(benzo[d]thiazol-2-yl)-2-aryl thiazolidin-4-one derivatives exhibited DPP4 inhibition with IC_50_ values at the nanomolar range, belonging to two classes: derivatives bearing a 6-OCF3-benzo[d]thiazol-2-yl moiety (compounds **h**) and derivatives bearing a 6-Ad-benzo[d]thiazol-2-yl (compound **m2**) or 4-CH_3_-6-Ad-benzo[d]thiazol-2-yl (compounds **n**) moiety.

Compounds in the **h** class exhibited higher potency. Compared to approved inhibitors [52], five compounds of the **h** class (**h1**, **h3**, **h4**, **h5**, and **h7**) exhibit lower IC_50_ values than vildagliptin (IC_50_: 62 nM) and saxagliptin (IC_50_: 50 nM), two lower than alogliptin (IC_50_: 24 nM) and one (**h3**) better than sitagliptin (IC_50_: 20 nM)

The **h3** compound, 3-(6-OCF_3_-benzo[d]thiazol-2-yl)-2-(2,6-2F-phenyl thiazolidin-4-one, of class **h** exhibited the best inhibition (IC_50_ = 12 ± 0.5 nM).

As confirmed by the IC_50_ values (Table 3), the substitution of the benzo[d]thiazol-2-yl moiety has the greatest effect in the inhibitory action with 6-OCF_3_ derivatives (**h**) being more potent than the 6-Ad (**m2**) and 4-CH_3_, 6-Ad (**n1/2**) derivatives, all exhibiting IC_50_ at the nM range. All 6-Cl derivatives (**c**) exhibited IC_50_ values at the μM range.

Five of the compounds of the **h**-group exhibited IC_50_ values 31 nM or lower, with compound **h3** showing IC_50_ = 12 ± 0.5 nM which is lower than the IC_50_ value of sitagliptin (20 ± 0.1 nM). In the h-group, the inhibitory action followed the sequence:

2-F, 6-Cl (**h3**, IC_50_ = 12 nM) > 2,4-di-Cl (**h5**) > 4-NO_2_ (**h7**) > 2,3-di-Cl (**h4**) > 2,6-di-Cl (**h1**, IC_50_ = 31 nM) > 4-OCH_3_ (**h9**) > 4-Cl (**h8**, IC_50_ = 90.6 nM) > 4-F (**h6**) and 4-OH (**h10**), with the last two being the less active compounds of the group with IC_50_ >100 nM.

Highlighting the effect of different halogen substituents, the replacement of the F atom at position 2 of the 2-F,6-Cl derivative **h3** (IC_50_ = 12 nM) with Cl leads to the less active 2,6-di-Cl derivative **h1** (IC_50_ = 31 nM). Although the presence of two Cl substituents at the two o-positions of the phenyl ring did not seem to be very favorable for inhibition, the location of two Cl at the o-, p- (**h5**) and o-, m- (**h4**) positions had better results with IC_50_ = 23.7 nM and 27 nM, respectively. Positioning of only one halogen at the p-position of the ring does not favor inhibition with the 4-Cl derivative (**h8**) exhibiting IC_50_ = 90.6 nM and 4-F derivative (**h6**) exhibiting IC_50_ > 100 nM. The 4-OCH_3_ derivative (**h9**, IC_50_ = 72.3 nM) had a little better activity than the 4-Cl, while the 4-OH derivative (**h10**) was less active with IC_50_ > 100 nM. Only the 4-NO_2_ derivative (**h7**) showed inhibitory action placing it among the five most active compounds of the **h**-group (IC_50_ = 25.5 nM).

Among the **m/n** group, compound **n2** (R1:4-CH_3_, 6-Ad, R2:2,6-di-F) showed the strongest inhibition with IC_50_ = 48.3 nM. The 2,6-di-Cl derivative (**n_1_**) had much lower inhibitory action with IC_50_ = 136.8 nM. According to the preliminary results of Table 2, the different halogens at the phenyl moiety of compounds **n1** and **n2** may cause radical changes in the binding of the inhibitors and inhibition mode with **n2** acting as competitive and **n1** probably acting as a non-competitive inhibitor. On the other hand, the adamantane derivative **m2** (R1: 6-Ad, R2: 2-F, 6-Cl) exhibits an intermediate IC_50_ = 71.5 nM but is also expected to differentiate at the inhibition mode probably exhibiting uncompetitive inhibition.

In the case of compounds of the **c**-group, which all have only one substituent at the p-position of the phenyl group, like in the less potent compounds of the **h**-group, this substitution may negatively contribute to the low activity of these compounds, in addition to the benzo[d]thiazol-2-yl substitution.

Interestingly, in accordance with the analog compounds of the **h**-group, a similar contribution of the kind of substituent in inhibition potency is observed. More precisely, the 4-NO_2_ derivative (**c2**) is more active with IC_50_ = 8.6 μM than the 4-F derivative (**c1**), which is the least active with IC_50_ > 20 μM, and the inhibitory action followed the sequence 4-NO_2_ (**c2**) > 4-OCH_3_ (**c4**) > 4-Cl (**c3**) > 4-F (**c1**). In the case of the **c**-group, **c4** is also expected to differentiate in the mode of inhibition probably acting as an uncompetitive inhibitor.

Kinetic experiments (Table 4, Figure 2) confirmed the existence of different kinds of inhibitors among compounds of both classes, among which **h3**, **h4**, **c2**, and **n2** exhibited competitive mode of inhibition, compounds **c4** and **m2** showed characteristics of uncompetitive inhibitors, and **n1** showed non-competitive inhibition profile. Compound **h9** showed a mixed inhibition mode.

A docking analysis performed for the estimation of the probable binding mode of the compounds indicated the thiazolidinone ring as a moiety of great importance for the orientation of the competitive inhibitors of both active classes (**h** and **m**) at the active center as it always interacts with Tyr666 and with Tyr547. In all cases, the benzo[d]thiazol-2-yl moiety is oriented towards Tyr585 and Gln553 with the benzene ring participating in hydrophobic and pi–pi interactions. The 6-OCF_3_ substituent of compounds **h** and the adamantane moiety of compound **n2** strongly contribute to complex stabilization by forming halogen bonds and hydrophobic interactions, respectively. The amino acids participating in these interactions are shown in Table 6.

The phenyl moiety at position 2 of the thiazolidinone is placed vertically to the benzo[d]thiazol-2-yl moiety (93^o^). In compounds of the h-group, this moiety is oriented towards Tyr662, towards Tyr547 and Ser630, or towards Glu205 and Glu206. This orientation seems to be influenced by the substituents of the phenyl ring and of the benzene ring of the benzo[d]thiazol-2-yl moiety (Table 7).

The Cl-atom at the o-position of the phenyl moiety, which favors activity, forms halogen bonds with Tyr662 (**h3**, **h5**), Tyr666 (**h3**), and Tyr547 (**h1**).

A F- (**h6**) or a -OH (**h10**) group at the p-position favors interactions with Ser630 and His740 leading to an analog orientation.

A Cl- (**h4**) at the m-position or a -NO_2_ (**h7**), -OCH_3_ (**h9**), or -Cl (**h8**) at the p-position leads to interactions with Glu206, and Ser209.

In the case of the adamantanyl derivative **n2**, the orientation of the phenyl ring within the active site is influenced by the methyl group at the 4-position of the benzene ring of the 3-(benzo[d]thiazol-2-yl) moiety and the halogens at the o-position of the phenyl ring. The methyl group participates in a hydrophobic interaction with Phe357 and Tyr547. This forces the molecule to turn the phenyl ring towards Glu205 and Glu206 with which the o-F atoms form halogen bonds.

In conclusion, according to our results, the 3-(benzo[d]thiazol-2-yl)-2-aryl thiazolidin-4-one offers a rigid, planar scaffold with groups at specific distances and angles which enables a specific mode of binding. In parallel, the benzo- and phenyl rings offer the opportunity for multiple hydrophobic and pi interactions, favoring orientation among the abundant aromatic amino acids of the specific areas of the active site.

**Figure 9:**
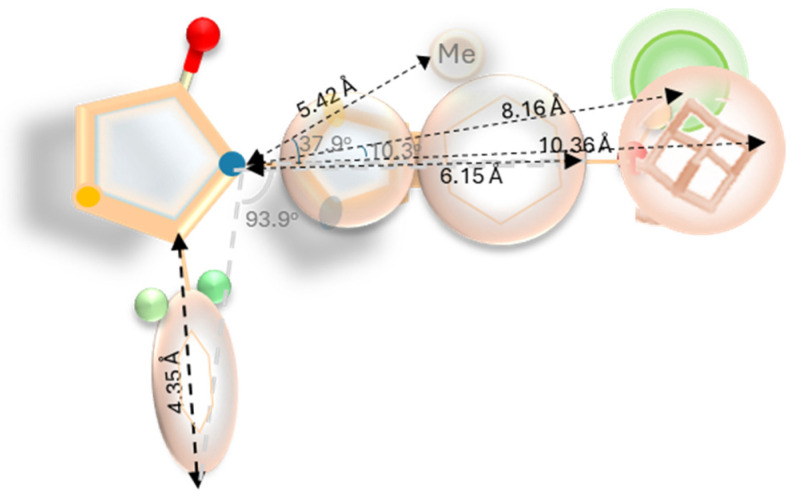
Favorable characteristics of competitive 3-(benzo[d]thiazol-2-yl)-2-aryl thiazolidin-4-one inhibitors. Green: halogens—participation in halogen bonds, Light brown spheres: groups participating in hydrophobic and pi–pi interactions. Blue: nitrogen, yellow: sulfur, red: oxygen.

A halogen(F)-rich group at a distance of 8.16 Å from the N of the thiazolidinone stabilizes the complex as proved by the activity of the compounds of group **h**. A Cl at the o-position of the phenyl group favors activity (Figure 9).

A second option is that provided by **n2**, where a highly hydrophobic, bulky moiety such as adamantane between 6.15 Å and 10.36 Å from the N of the thiazolidinone stabilizes the orientation of the main skeleton. A hydrophobic group at 5.42 Å from the N of the thiazolidinone and at 37.9° from the main axon of benzo[d]thiazol-2-yl moiety, in collaboration with the two F- atoms at the o-position of the phenyl ring, assures the right final orientation of the inhibitor (Figure 9).

Compared to the studied compounds, approved competitive inhibitors such as sitagliptin, alogliptin, and saxagliptin, are more polar molecules, containing a greater number of nitrogen and oxygen atoms which participate in hydrogen bonds with a greater share in complex stabilization. Despite the presence of hydrophobic and/or aromatic moieties in the molecules, their contribution is less strong.

On the contrary, aromatic, hydrophobic groups and halogen atoms mostly participate in the complex stabilization of the studied compounds. The length and structure of the compounds enable interactions with the aromatic/hydrophobic amino acid-rich regions of the active site.

Although the existence of covalent bonds are not observed in the crystallographic structures of sitagliptin (PDB: 4FFW), alogliptin (PDB: 3G0B), or saxagliptin (PDB: 6B1O) formation of such bonds have been mentioned in the literature for the cyanopyrrolidines such as saxagliptin and vildagliptin where the -C≡N group is believed to form reversible covalent bonds with Ser630 [53]. A covalent reversible bond with Ser630 has also been referred to for Sitagliptin.

The studied compounds do not possess the -C≡N group thus avoiding covalent bond formation. Apart from the probability of undesired prolonged inhibition of DPP4 with the risk of an abnormal increase in specific substrates, irreversible covalent bonds with cysteines have been referred recently between the -C≡N group and other proteins, conferring to the side effects of these compounds [54].

Among the most interesting findings in this study was the presence of inhibitors with uncompetitive and non-competitive modes of action. Interestingly, the three representatives of the adamantane class exhibit competitive (**n2**), non-competitive (**n1**), and uncompetitive (**m2**) inhibitory action.

The three structures differ in the -CH_3_ substituent of the benzene ring of the benzo[d]thiazol-2-yl moiety and in the halogens of the o-position of the phenyl ring.

**Figure 10:**
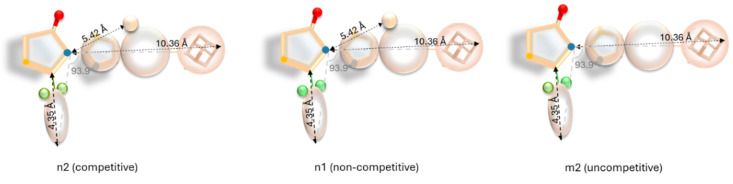
Characteristics of adamantane derivatives with competitive, non-competitive, and uncompetitive modes of inhibition.

The presence of the methyl group and two F-substituents leads to competitive inhibitor, the presence of the methyl group and two Cl-substituents directs to non-competitive inhibition, while the absence of the methyl group and one Cl- and one F-substituent leads to uncompetitive inhibition (Figure 10).

According to the docking results, all three compounds can bind at the active site with good Eest. What mostly differentiates the modes of inhibition is their ability to bind more strongly to other sites apart from the active site.

The low number of compounds with non-competitive and uncompetitive inhibition does not enable safe conclusions about the structure which could favor these modes of action. However, the results encourage further study of derivatives of this class as well as crystallographic studies for binding site identification.

The effort for the estimation of probable allosteric sites for non-competitive or uncompetitive inhibitors using docking analysis indicated a probable site for each kind of inhibitor in the wide area of the active site and a second binding site for uncompetitive inhibitors in the propeller loop. The estimation was based on the absence of binding of other inhibitors at the specific sites, the ranking, and the estimated energy of binding to the specific sites. Calculation of the Probability Factor (PF) for binding of the non-competitive inhibitor at the probable site revealed a good correlation with the determined IC_50_ value (Figure 4). Calculation of the Probability Factor for binding of uncompetitive inhibitors to both candidate allosteric sites revealed that, in both cases, the PF correlated well with logIC_50_, thus indicating a similar probability of binding to both sites. Apparently, only crystallographic and NMR studies can answer the question.

Apart from its usefulness in the estimation of probable binding sites of allosteric inhibitors, the calculation of the Probability Factor, PF, may facilitate the prediction of IC_50_ values of potential inhibitors.

The results in this study indicated that the determined IC_50_ values of the competitive inhibitors followed (R^2^ = 0.9881, *p* = 0.00005347) the following equation well:

logIC_50_ = 0.6704824 ∗ PF + 6.451999

where: **PF = Eest_ts_ − {**$\sum \left[({Eest}_{x}-{Eest}_{t})*\frac{vx}{100}\right]$ ∗ 10},

Eest_ts_: Estimated Binding Energy to the target site when the docking is applied to the target site box.
Eest_t_: Estimated Binding Energy to the target site when docking is applied to the whole enzyme.
Eest_x_: Estimated Binding Energy to the site x lower than Eest_t_ when the docking is applied to the whole enzyme.
v_x_: frequency % of the docking pose with Estimated binding Energy Eest_x_

As the most potent compounds could be considered as drug candidates for the treatment of Diabetes type II, the potential toxicity of the compounds must be discussed.

Most toxicity effects of approved DPP-4 inhibitors are attributed to abnormal increases in their substrates probably due to the increased/prolonged activity of the inhibitors at the local or general level.

An increase in the concentration of the DPP4 substrates GLP-1, GIP (also called Gastric Inhibitory Polypeptide), the Pituitary Adenylate Cyclase-Activating Polypeptide (PACAP) and oxyntomoduline has been related to gastrointestinal disorders [17,55]. The side effects are believed to be due to the gastrointestinal actions of the peptides apart from their Insulinotropic activity.

Acute pancreatitis, a rare but severe side effect of DPP4 inhibitors, was associated with increased activity of GLP-1, which can cause ductal hyperplasia in the pancreas with outflow obstruction leading to intrapancreatic activation of pancreatic enzymes [18,56].

The mechanism proposed for increased incidences of cardiac failure hospitalization was related to elevated concentration of the DPP4 substrates, substance P and Neuropeptide Y, both of which increase heart rate by increasing sympathetic activity. The incidences were reduced by the concomitant administration of B-blockers or ACE inhibitors which reduce blood pressure [19].

Interestingly, DPP4 inhibitors such as sitagliptin and alogliptin which are mainly excreted in the urine in their initial, unmodified form were not associated with this side effect. The inhibitors decrease the action of the sodium-hydrogen exchanger 3 (NHE3), involved in sodium reabsorption, thus decreasing blood sodium levels and blood pressure. NHE3 activity is related to DPP4 with which it forms complex, while increased expression of the exchanger is related to diabetes and heart failure [19].

The increase in substance P levels can also explain Myalgia, by its effect on the pain threshold [17].

On the other hand, an increase in inflammation mainly of the respiratory and urinary tract is related to the direct effect of the enzyme in T lymphocyte regulation and the suppression of mitogen-stimulated T-cell responses by DPP-4 inhibitors [17].

It is obvious that there is a drug-class-related organ-specific toxicity, although differences between specific drugs exist mainly attributed to differences in drug kinetics.

Cytotoxicity experiments conducted for some compounds of this seria using MRC-5 normal cells showed no evidence of cytotoxicity at a compound concentration of 10 μM [44]. However, cytotoxicity was not evaluated for all studied compounds.

Since class-related, organ-specific toxicity is observed, involving peptides with paracrine or endocrine activity, cytotoxicity experiments cannot give reliable results, even if organ-specific cell cultures and specific toxicity measuring parameters are used. In vivo experiments using laboratory animals give a better picture.

Computational programs can give a prediction based on the structure relationship with known compounds along with all the restrictions accompanying these methods.

According to the prediction results, Table 5, all compounds are expected to exhibit permeability and human intestinal absorption (HIA: 88.09–93.05%) analog or better than the approved drugs, linagliptin, sitagliptin, alogliptin, Saxagliptin, and Vildagliptin, which were used for comparison reasons.

Except for compound **h6**, they all exhibit LD_50_ values analogous to or better than the approved inhibitors, varying between 2.416 and 3.047 mol/Kg.

Eleven out of the sixteen compounds were predicted not to exhibit hepatotoxicity, cardiotoxicity, nephrotoxicity, or carcinogenicity. For compounds **c2** and **h7**, nephrotoxicity and carcinogenicity were predicted, while hepatotoxicity was predicted for compounds **h6** and **h7** with a weak probability of 0.50–0.54. For the most active compounds, no organ-specific toxicity was predicted.

Hepatotoxicity was predicted for the approved drugs with the exception of vildagliptin.

Hepatic injury attributed to gliptins has been mentioned [54,57]. This has not been confirmed by clinical trials, although in vivo results support toxicity, elucidating the mechanism underneath this effect for saxagliptin attributing the side effect to the capability of the drug to form irreversible covalent bonds with cysteine residues of endogenous proteins [54]. No cardiotoxicity was predicted for the approved drugs, probably due to the low proportion of the incidences among the recorded side effects.

Metabolism prediction results indicated a probable involvement of CYP3A4 in the metabolic process of all the compounds and no involvement of CYP2D6. The prediction profile is similar to that of linagliptin and alogliptin. On the contrary, sitagliptin and vildagliptin are not expected to act as substrates of these enzymes, while both enzymes are expected to be involved in saxagliptin metabolism (Table 5).

The results concerning approved inhibitors are partially in accordance with in vivo results. According to the literature, saxagliptin and linagliptin are metabolized by CYP3A4 although 87% of linagliptin is excreted unmodified in the feces. Allogliptin is metabolized to a low extent with CYP3A4 and CYP2D6 being the two cytochrome enzymes involved in its metabolism. CYP3A4 also slightly contributes to sitagliptin metabolism, while 79% of the drug ends unmodified in the urine. Vildagliptin is mainly metabolized by renal enzymes leading to drug Inactivation [58,59,60,61].

## 4. Methods

### 4.1. In Vitro DPP4 Inhibition Assay [62]

DPP-4 activity was assessed by measuring the hydrolysis rate of the substrate H-Gly-Pro-amidomethyl coumarin. The resulting fluorescent product, 7-Amino-4-Methyl Coumarin (AMC), was monitored with a Tecan Pro2000 microplate fluorescence reader (Tecan Ltd, Zurich, Switzerland) at an excitation wavelength of 360 nm and an emission wavelength of 460 nm, with measurements taken every 60 s for 30 min. The inhibitors, dissolved in DMSO, were added to the reaction buffer (20 mM Tris-HCl, pH 8, 100 mM NaCl, and 1 mM EDTA) at 10 µL per reaction, along with 0.17 µg/mL of enzyme, and incubated for 10 min. After incubation, 50 µL of the appropriate substrate concentration was added, bringing the total reaction volume to 100 µL. The inhibition (%) was calculated as ((Fc − Fi)/Fc) × 100, where Fc is the fluorescence value of the control (no inhibitor-total activity) and Fi is the fluorescence value in the presence of the inhibitor. The IC_50_ values were calculated from the inhibition % vs. log [Inhibitor] plot at Graphpad Prism 10.3.1. The volume of 10 μL DMSO was added to the total activity control instead of the inhibitor. A blank containing all other components except for the enzyme was abstracted in all cases. Each determination was performed in triplicate.

### 4.2. Inhibition Kinetic Analysis of DPP4

Selected compounds with higher inhibitory activity or compounds that exhibited characteristics of other inhibitory types except competitive, were used to perform inhibition kinetic analysis. The concentration range of the substrate (H-Gly-Pro-AMC) was 17–80 μM, and the final concentration of DPP-4 was 0.17 µg/mL. The maximum enzymatic reaction rate (Vmax) and Michaelis constant (Km) were calculated according to the Lineweaver–Burk plot. The inhibition types (competitive, uncompetitive, non-competitive, or mixed) of the tested compounds were determined by using Lineweaver–Burk plot, which showed ΔF^−1^min vs. [S]^−1^.

### 4.3. Methodology of Molecular Docking 

Docking analysis was performed on the molecular docking server using Autodock 4 [63,64], as previously described [49]. For the docking simulation, the Lamarckian genetic algorithm (LGA) and the Solis and Wets local search method [65] were used. During docking, all rotatable torsions were released. AutoDock parameter set- and distance-dependent dielectric functions were used in the calculation of the van der Waals and the electrostatic terms, respectively—100 different runs that were set to terminate after a maximum of 2,500,000 energy evaluations were performed in each docking experiment. The population size was set to 150. During the search, a translational step of 0.2 Å, and quaternion and torsion steps of 5 were applied. The estimated free binding energy, calculated by the program, is an indicator of the probability of the compounds forming a stable complex with the selected enzyme target and consequently the probability of being effective enzyme inhibitors.

The protein structures chosen from the Protein Data Bank and the docking center and docking box chosen for each docking analysis were as follows: for docking analysis of the DPP4, the structure 2OAG of the enzyme in a complex with the competitive inhibitor (3R,4S)-1-{6-[3-(methylsulfonyl)phenyl]pyrimidin-4-yl}-4-(2,4,5-trifluorophenyl) pyrrolidin-3-amine (DLI B) [66] was used. The docking center was kept at x = 34.82, y = 5.53, and z = 63.51 and the docking box was set at x = 20, y = 20, and z = 20 to surround the binding site of the inhibitor (active site of the enzyme). The docking box was extended to x = 100, y = 100, and z = 100 to include all the enzyme for docking analysis for the whole enzyme. For standard docking analysis, the initial ligand was abstracted during the preparation process. For the non-competitive and uncompetitive inhibitors, a third docking application was performed to the whole enzyme containing the initial inhibitor.

**Figure 11:**
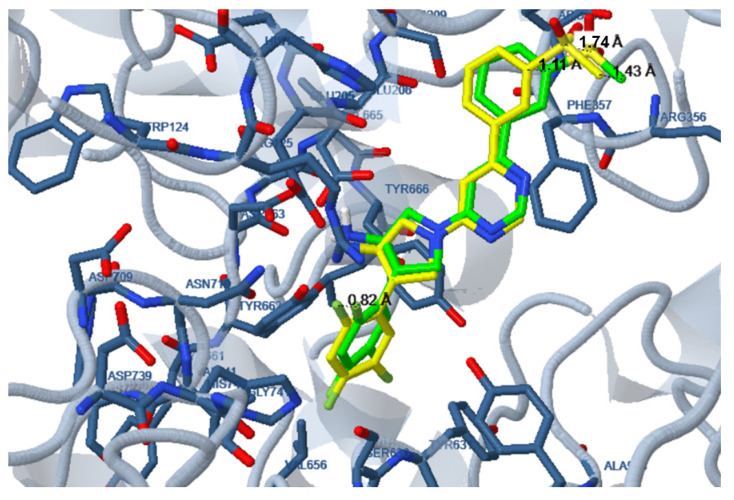
Verification process. Docking of the initial ligand (DLI B) to the enzyme (PDB:2OAG) from which the ligand was abstracted. Docked ligand is shown in green. The position of the initial ligand is shown in yellow. Indicative distances between the same atoms of the initial and docked ligand are shown.

Before application to the studied compounds, a verification process was conducted by docking the initial ligand to the enzyme from which the ligand was abstracted and comparison of the position of the docked ligand with that of the initial ligand. The position of the docked ligand was very similar to that of the initial ligand with the maximum distance between identical atoms of the initial and docked ligand not exceeding 2 Å (Figure 11).

### 4.4. Toxicity Prediction

Toxicity Prediction was performed using the pkCSM Program [67] and the ProTox-3 Program [68]. The pkCSM program predicts Small-Molecule Pharmacokinetic and Toxicity Properties using Graph-Based Signatures. ProTox 3.0 predicts toxicity of small molecules incorporating molecular similarity, fragment propensities, most frequent features, and (fragment similarity-based CLUSTER cross-validation) machine-learning, based on a total of 61 models for the prediction of toxicity endpoints such as acute toxicity, organ toxicity, toxicological endpoints, molecular initiating events, metabolism, adverse outcomes (Tox21) pathways, and toxicity targets.

## 5. Conclusions

Twelve out of the sixteen 3-(benzo[d]thiazol-2-yl)-2-aryl thiazolidin-4-one derivatives which were initially selected for in vitro evaluation exhibited DPP4 inhibition with IC_50_ value at the nM range. Two classes of the tested compounds gave the best results: the 6-OCF_3_- and the 6-Ad- or 4CH_3_,6-Ad- benzo[d]thiazol-2-yl derivatives (classes **h**, **m/n**). The best inhibitor, 3-(6-OCF3-benzo[d]thiazol-2-yl)-2-(2-F,6-Cl-phenyl) thiazolidin-4-one (**h3**), showed an IC_50_ value of 12 ± 0.5 nM, better than the IC_50_ value of sitagliptin (20 ± 0.1 nM).

Most of the compounds exhibited a competitive mode of inhibition with no covalent bond formation. However, inhibition modes of the uncompetitive and non-competitive types, which are not common in DPP4 inhibitors, were identified for some of the compounds. The 3-(6-Ad-benzo[d]thiazol-2-yl)-2-(2-F,6-Cl-phenyl) thiazolidin-4-one was the best uncompetitive inhibitor with IC_50_ = 71.5 ± 8.3 nM while the 3-(4-CH_3_,6-Ad-benzo[d]thiazol-2-yl)-2-(2,6-di-Cl-phenyl) thiazolidin-4-one exhibited non-competitive inhibition with IC_50_ = 136.8 ± 4.6 nM. Although non-competitive and uncompetitive inhibitors have lower activity, they may be of interest, since such inhibitors could cause more balanced inhibition concerning substrate concentrations. Moreover, the discovery of such inhibitors may constitute the first step for finding of allosteric sites of the enzyme, better exploring its role and interactions.

Docking analysis is in accordance with the in vitro results, with a linear correlation of logIC_50_ with a Probability of Binding Factor, PF, derived using results from docking to a specific target box and to the whole enzyme.

Docking analysis used for the estimation of binding mode indicated the probable binding of the compounds. Two sites in the wide area of the active site and at the propeller loop were the most probable for uncompetitive inhibitors. However, crystallographic and NMR studies are needed to elucidate the exact way of binding especially for uncompetitive and non-competitive inhibitors.

## Figures and Tables

**Table 1:** Estimated Binding Energy of the compounds at the active site and at the Whole enzyme.

N	Est. Binding Energy in DPP4 Active Site (kcal/mol)	Est. Binding Energy in Whole DPP4 (kcal/mol)	N	Est. Binding Energy in DPP4 Active Site (kcal/mol)	Est. Binding Energy in Whole DPP4 (kcal/mol)
**h1**	−8.09	−7.95	**h10**	−6.94	−6.78
**h3**	−8.32	−9.11	**m2**	−9.64	−11.04
**h4**	−8.09	−8.84	**n1**	−9.81	−9.42
**h5**	−8.75	−8.65	**n2**	−9.05	−9.61
**h6**	−7.80	−8.52	**c1**	−6.96	−8.52
**h7**	−8.92	−9.05	**c2**	−7.23	−8.35
**h8**	−8.65	−8.96	**c3**	−7.50	−8.96
**h9**	−7.80	−8.16	**c4**	−6.63	−7.50

**Table 2:** Percentage of inhibition at two substrate concentrations.

Compound	Compound Concentration (μM)	% Inhibition	
		**50 μΜ Substrate**	**100 μΜ Substrate**
**c1**	10	33.5	11.4
**c2**	10	66.0	14.1
**c3**	10	41.6	28.0
**c4**	10	**48.5**	**68.5**
**h1**	0.080	79.8	12.0
**h3**	0.080	62.0	20.0
**h4**	0.080	84.4	12.1
**h5**	0.080	45.0	14.0
**h6**	0.080	46.8	0.0
**h7**	0.080	67.0	55.0
**h8**	0.080	45.0	26.0
**h9**	0.080	52.3	37.6
**h10**	0.080	38.0	0.0
**m2**	0.080	**52.0**	**69.7**
**n1**	0.080	**36.8**	**37.2**
**n2**	0.080	66.3	0.0

**Table 3:** IC_50_ values of the compounds.

Compound	IC_50_	Compound	IC_50_
**h1**	31 ± 3.8 nM	**m2**	71.5 ± 8.3 nM
**h3**	12 ± 0.5 nM	**n1**	136.8 ± 4.6 nM
**h4**	27 ± 0.5 nM	**n2**	48.3 ± 5.2 nM
**h5**	23.7 ± 2 nM	**c1**	>20μΜ
**h6**	>100 nM	**c2**	8.6 ± 0.2 μM
**h7**	25.5 ± 3 nM	**c3**	12.5 ± 0.2 μΜ
**h8**	90.6 ± 8.8 nM	**c4**	11.5 ± 1.5 μΜ
**h9**	72.3 ± 5.2 nM		
**h10**	>100 nM	**sitagliptin**	20 ± 0.1 nM

**Table 4:** Km and Vmax values of the enzyme in the presence of the compounds and inhibition mode.

Compound	Concentration (μΜ)	Κm	Vmax	Inhibition Mode
**-**	0	49.7 ± 3.8	1085.7 ± 57.4	-
**h3**	0.010	103.1 ± 9.3	1019.0 ± 9.7	competitive
**h4**	0.080	98 ± 26.9	1021 ± 36.4	competitive
**h8**	0.080	167.9 ± 15.8	839.2 ± 98.9	mixed
**m2**	0.060	30.6 ± 2.7	599.0 ± 50.8	uncompetitive
**n1**	0.100	49.8 ± 0.6	772.8 ± 2.5	non-competitive
**n2**	0.100	117.2 ± 1.5	1112.1 ± 48.5	competitive
**c2**	15.000	87.6 ± 4.8	1055.5 ± 16.1	competitive
**c4**	15.000	19.4 ± 9.3	342.0 ± 81.1	uncompetitive

**Table 5:** Prediction of absorption, metabolism, and toxicity of the compounds.

Comp.	LD_50_ ^1^ (mol/kg)	Perm ^1^	HIA ^1^ %	Organ-Specific Toxicity				Metabolism ^1^	
				Hep.^1^	Card.^2^	Nephr.^2^	Carc.^2^	CYP2D6 Substr.	CYP3A4 Substr.
**c1**	2.667	1.541	92.13	No	Νο	No	No *	No	Yes
**c2**	2.833	1.178	91.87	No	No	Yes *	Yes *	No	Yes
**c3**	2.727	1.120	91.23	No	No	No	No *	No	Yes
**c4**	2.725	1.134	93.05	No	No	No	No *	No	Yes
**h1**	2.994	1.112	89.47	No	No	No	No *	No	Yes
**h3**	3.213	1.164	90.42	No	No	No	No *	No	Yes
**h4**	3.019	1.121	89.50	No	No	No	No *	No	Yes
**h5**	2.976	1.115	88.88	No	No	No	No *	No	Yes
**h6**	0.623	1.153	91.61	Yes	No	No	No *	No	Yes
**h7**	2.416	1.208	88.09	Yes	No	Yes *	Yes *	No	Yes
**h8**	2.885	1.119	90.54	No	No	No	No *	No	Yes
**h9**	2.893	1.174	91.22	Yes	No	No	No *	No	Yes
**h10**	2.681	1.132	89.56	Yes	No	No *	No	No	Yes
**m2**	3.047	1.028	91.48	No	No	No	No *	No	Yes
**n1**	2.950	1.110	90.44	No	No	No	No *	No	Yes
**n2**	2.899	1.184	92.32	No	No	No	No *	No	Yes
**Linagl**	2.620	0.356	83.98	Yes	No	No	No *	No	Yes
**Sitagl.**	2.974	1.245	89.39	Yes	No	No	No *	No	No
**Alogl.**	2.421	0.175	79.50	Yes	No	No	No	No	Yes
**Saxagl**	2.500	0.614	81.36	Yes	No	No	No	Yes	Yes
**Vildagl**	2.674	0.591	69.81	No	No	No *	No	No	No

^1^ pkcsm program, ^2^ ProTox 3.0 Program, Perm.: Caco2 permeability (logPapp in 10^−6^ cm/s), HIA: Human Intestinal Absorption, Hep.: Hepatotoxicity, Card.: Cardiotoxicity, Nephr.: Nephrotoxicity, Carc.: Carcinogenicity, Linagl: Linagliptin, Sitagl.: Sitagliptin, Alogl. Alogliptin, Saxagl.: Saxagliptin, Vildagl: Vildagliptin, * probability 0.5–0.59. In all other cases, the probability is 0.60–0.91.

**Table 6:** Amino acids participating in interactions with competitive inhibitors.

	Thiazolidine-4-one	Thiazolyl-	Benzo-	-OCF_3_ (hal)	Ad (Hph)	Phenyl Ring
* h3 (2-F, 6-Cl)	Tyr547(Hb), Tyr666(Hph)	Phe357(pi)	Phe357(pi) Tyr547(pi)	Tyr585 Cys551	-	
**h5** (2,4-di-Cl)		Tyr547(Hb)	Phe357(pi) Tyr547(pi)	Gln553	-	His740
**h7** (4-NO_2_)	Tyr547(pol) Arg125(pol) Tyr666(Hph)	Tyr547(Hb)	Phe357(pi) Tyr547(pi)	Tyr585 Cys551	-	Phe357(pi) Tyr666(pi)
**h4** (2,3-di-Cl)	Tyr547(pol) Tyr666(pol, Hph)	Tyr547(Hb)	Phe357(pi) Tyr547(pi)	Gln553	-	Phe357(pi) Tyr666(pi)
**h1** (2,6-di-Cl)		Tyr547(Hb) Phe357(pi)	Phe357(pi) Tyr547(pi)	Tyr585 Cys551	-	Tyr666(pi)
**h9** (4-OCH_3_)	Tyr547(pol) Arg125(pol) Tyr666(Hph)	Tyr547(Hb)	Phe357(pi) Phe547(pi)	Gln553	-	Phe357(pi) Tyr666(pi)
**h8** (4-Cl)	Tyr547(pol) Arg125(pol) Tyr666(Hph)	Tyr547(Hb)	Phe357(pi) Phe547(pi)	Tyr585 Cys551	-	Phe357(pi) Tyr666(pi)
**h6** (4-F)	Tyr547(pol) Tyr666(Hph)	Tyr547(Hb)	Phe357(pi) Phe547(pi)	Gln553	-	His740(pi)
**h10** (4-OH)	Tyr547(Hb, pol) Arg125(pol) Tyr666(Hph)		Phe357(pi) Phe547(pi	Gln553	-	His740(pi)
**n2**	Tyr547(pol) Tyr666(Hph)	Tyr547(Hb)	Phe357(pi) Phe547(pi)	-	Tyr585	

* Compound ranking in decreasing activity, Hb: Hydrogen bond, Hph: Hydrophobic interaction, pi: pi–pi interactions, hal: halogen bond, pol: polar interaction.

**Table 7:** Interaction of the substituents of the phenyl moiety in compounds of class **h**.

	o-Cl	m-X	p-X
* h3 (2-F, 6-Cl)	Tyr662, Tyr666	-	-
**h5** (2,4-di-Cl)	Tyr662	-	-
**h7** (4-NO_2_)	-	-	Glu206 (Hb), Ser209 (Hb)
**h4** (2,3-di-Cl)	-	Ser209	-
**h1** (2,6-di-Cl)	Tyr547	-	-
**h9** (4-OCH_3_)	-	-	Ser209 (Hb), Phe357 (Hph)
**h8** (4-Cl)	-	-	Glu206 (Hal), Ser209 (Hal)
**h6** (4-F)	-	-	Ser630 (Hal)
**h10** (4-OH)	-	-	Ser630 (pol), His740 (pol)

* Compound ranking in decreasing activity, Hb: Hydrogen bond, Hph: Hydrophobic interaction, Hal: halogen bond, pol: polar interaction.

## Data Availability

The raw data supporting the conclusions of this article will be made available by the authors on request.
